# *Nicotiana benthamiana* RanBP1-1 Is Involved in the Induction of Disease Resistance via Regulation of Nuclear-Cytoplasmic Transport of Small GTPase Ran

**DOI:** 10.3389/fpls.2019.00222

**Published:** 2019-03-08

**Authors:** Yuri Mizuno, Mina Ohtsu, Yusuke Shibata, Aiko Tanaka, Maurizio Camagna, Makoto Ojika, Hitoshi Mori, Ikuo Sato, Sotaro Chiba, Kazuhito Kawakita, Daigo Takemoto

**Affiliations:** Graduate School of Bioagricultural Sciences, Nagoya University, Nagoya, Japan

**Keywords:** *Nicotiana benthamiana*, nucleoporin, *Phytophthora infestans*, Ran binding protein1, capsidiol

## Abstract

Plant cells enhance the tolerances to abiotic and biotic stresses via recognition of the stress, activation and nuclear import of signaling factors, up-regulation of defense genes, nuclear export of mRNA and translation of defense proteins. Nuclear pore-mediated transports should play critical roles in these processes, however, the regulatory mechanisms of nuclear-cytoplasmic transport during stress responses are largely unknown. In this study, a regulator of nuclear export of RNA and proteins, NbRanBP1-1 (Ran-binding protein1-1), was identified as an essential gene for the resistance of *Nicotiana benthamiana* to potato blight pathogen *Phytophthora infestans*. *NbRanBP1-1*-silenced plants showed delayed accumulation of capsidiol, a sesquiterpenoid phytoalexin, in response to elicitor treatment, and reduced resistance to *P. infestans*. Abnormal accumulation of mRNA was observed in *NbRanBP1-1*-silenced plants, indicating that NbRanBP1-1 is involved in the nuclear export of mRNA. In *NbRanBP1-1*-silenced plants, elicitor-induced expression of defense genes, *NbEAS* and *NbWIPK*, was not affected in the early stage of defense induction, but the accumulation of NbWIPK protein was reduced. Nuclear export of the small G-protein NbRan1a was activated during the induction of plant defense, whereas this process was compromised in *NbRanBP1-1*-silenced plants. Silencing of genes encoding the nuclear pore proteins, *Nup75* and *Nup160*, also caused abnormal nuclear accumulation of mRNA, defects in the nuclear export of NbRan1a, and reduced production of capsidiol, resulting in decreased resistance to *P. infestans*. These results suggest that nuclear export of NbRan is a key event for defense induction in *N. benthamiana*, and both RanBP1-1 and nucleoporins play important roles in the process.

## Introduction

Solanaceae plants include many economically important crops such as *Solanum* (potato, tomato, and eggplant), *Capsicum* (chili and bell peppers), and *Nicotiana* (tobacco) species. Among them, potato is one of the major staple foods for many countries all over the world. One critical problem for potato production is the control of late blight disease caused by the oomycete pathogen *Phytophthora infestans*. Potato late blight is known as a causal agent of the Irish “Great famine” in the 1840s, and even today, annual cost for yield losses and control efforts in developing countries are estimated around 9 billion euro (Haverkort et al., 2009). The resistance of potato cultivars against *P. infestans* is generally determined by dominant resistance (*R*) genes encoding nucleotide-binding site leucine-rich repeat (NBS-LRR) type proteins involved in the recognition of attempted pathogen infection. So far, over 20 *R* genes conferring race-specific (e.g., *R1* and *R2*), intermediate spectrum (e.g., *R2* and *Rpi-blb3*), or broad spectrum (e.g., *Rpi-blb1* and *Rpi-vnt1.1*) resistance have been identified from several *Solanum* species, and large efforts have been taken to introduce broad-spectrum *R* genes into cultivated potato to achieve durable resistance to late blight (Haverkort et al., 2016). However, the underlying molecular mechanisms for the activation and execution of *R* gene-dependent resistance of potato is still largely unknown.

The Solanaceae plant, *Nicotiana benthamiana* has been extensively utilized as a model plant, because techniques such as *Agrobacterium*-mediated transient expression (so-called Agroinfiltration) and virus-induced gene silencing (VIGS) are well established for this plant (Ratcliff et al., 2001; Goodin et al., 2008). There are no reports of any naturally occurring disease of *Nicotiana* plants by infection of *P. infestans*, and it was shown that mature *N. benthamiana* is resistant to inoculation with *P. infestans* isolates (Shibata et al., 2011). This makes *N. benthamiana* an ideal model plant for investigating Solanaceae genes required for resistance to *P. infestans*.

We have taken VIGS-based forward and reverse genetics approaches to isolate *N. benthamiana* genes required for resistance to *P. infestans* (Shibata et al., 2010, 2011, 2016; Matsukawa et al., 2013; Ohtsu et al., 2014; Takemoto et al., 2018). The major groups of genes isolated from our screening are enzymatic genes of the mevalonate and ethylene production pathways (Shibata et al., 2016). The main phytoalexin of *Nicotiana* species is capsidiol, a sesquiterpenoid derived from the mevalonate pathway (Bailey et al., 1975; Takemoto et al., 2018). Genes encoding enzymes for production of farnesyl pyrophosphate and the following two steps catalyzed by *NbEAS* (5-*epi*-aristolochene synthase) and *NbEAH* (5-*epi*-aristolochene dihydroxylase) are required for capsidiol production as well as resistance to *P. infestans*, indicating that production of the capsidiol plays a principal role in resistance of *N. benthamiana* to *P. infestans* (Shibata et al., 2010, 2016). PDR-type ABC transporters NbABCG1 and NbABCG2 have been identified as the exporters of capsidiol, which contribute to resistance against *P. infestans* by targeted secretion of capsidiol at the site of pathogen attack (Shibata et al., 2016).

Ethylene signaling is the key regulator of phytoalexin production in N. benthamiana. NbEIN2, the central regulator of ethylene signaling, is required for elicitor-induced expression of NbEAS, NbEAH, and NbABCG1/2 genes, thus ethylene is involved in the production and secretion of capsidiol during pathogen attack (Shibata et al., 2010, 2016). Several genes for ethylene biosynthesis also have been isolated as essential genes for the resistance of N. benthamiana by VIGS-based forward genetics screening, further confirming that ethylene is crucial for inducing disease resistance in N. benthamiana (Shibata et al., 2016). Ethylene production is under the control of a MAP (mitogen-activated protein) kinase cascade, namely NbMEK2-NbWIPK/SIPK in N. benthamiana. Gene silencing of NbWIPK/NbSIPK (wound-induced/salicylic acid-induced protein kinase) caused significant reduction of ethylene production during plant defense, which resulted in reduced capsidiol production and resistance to P. infestans (Ishihama et al., 2011; Ohtsu et al., 2014). In Arabidopsis, expression of AtACS2 and AtACS6, encoding key enzymes for ethylene production, are regulated by the AtMKK4/MKK5-AtMPK3/MPK6 cascade, an orthologous MAPK cascade of N. benthamiana NbMEK2-NbWIPK/SIPK (Liu and Zhang, 2004; Han et al., 2010).

The gene for nuclear pore complex (NPC) protein NbNup75 (Nucleoporin 75) has been identified as a required component for the resistance to *P. infestans* (Ohtsu et al., 2014). At least 30 nucleoporins, components of the NPC, were identified in Arabidopsis and *N. benthamiana* (Tamura et al., 2010; Ohtsu et al., 2014), and Nup75 is a member of Nup107-160 subcomplex, a component of the nuclear pore scaffold. Gene silencing of *NbNup75* has no significant effect on phosphorylation of NbSIPK and NbWIPK and induction of defense genes in the early stage of defense induction, while induction of resistance reaction in the later stage such as ethylene and phytoalexin production was suppressed in *NbNup75*-silenced plants. Therefore, it was indicated that Nup75 is not involved in the initial signal transduction from recognition of elicitor to the up-regulation of defense genes, which instead is probably mediated by preexisting signaling factors.

The NPC constitutes the gate on the nuclear envelope which mediates the nucleo-cytoplasmic transport of cargo, such as RNAs and proteins (Tamura et al., 2010). In NbNup75-silenced N. benthamiana, cellular distribution of NbSIPK and NbWIPK was not affected, while the abnormal accumulation of mRNA in nuclei was observed (Ohtsu et al., 2014). These results indicated that NbNup75 is required for the efficient export of mRNA, which probably is a critical process for timely production of defense-related proteins. Defects in nuclear export of mRNAs and plant defense also have been reported in Arabidopsis mutants of the Nup107-160 subcomplex (Wiermer et al., 2012). In Lotus japonicus, mutations in the nucleoporin genes Nup85 (the ortholog of NbNup75), Nup133, and Seh1, lead to defects in plant-microbe symbiotic interactions (Kanamori et al., 2006; Saito et al., 2007; Groth et al., 2010).

In this study, our forward genetic screening using VIGS-mediated random gene silencing identified a gene encoding Ran-binding protein1 (*NbRanBP1-1a*) as an essential gene for disease resistance of *N. benthamiana* to *P. infestans*. RanBP1 is known as a regulator of nucleo-cytoplasmic trafficking, which controls the cargos transport across nuclear envelope. In yeast, RanBP1 localizes to cytoplasmic filaments of NPC and is involved in the release of exported cargos from nuclei via hydrolysis of GTP-bound Ran, the master regulator of nucleo-cytoplasmic transport (Kehlenbach et al., 1999). We examined the intracellular distribution of mRNA, expression of defense genes and phytoalexin production in *NbRanBP1-1*-silenced plants to dissect the role of NbRanBP1-1 in the induction of plant defense responses.

## Materials and Methods

### Biological Materials, Growth Conditions, and Inoculation

*N. benthamiana* (line SNPB-A5, Shibata et al., 2016) or *N. tabacum* (cv. Samsun NN) were grown in an environmentally controlled growth room at 23°C under a 16 h of light/8 h dark per day. *P. infestans* isolate 08YD1 (Shibata et al., 2011) was cultured on rye media at 20°C. Inoculation of *N. benthamiana* leaves (˜45 days old) with a zoospore suspension of *P. infestans* was performed as described previously (Shibata et al., 2010).

### Preparation and Treatment of *N. benthamiana* Leaves With INF1 Elicitor

INF1 elicitor was prepared from *Escherichia coli* (DH5α) carrying an expression vector for INF1, pFB53 as previously reported (Kamoun et al., 1997; Shibata et al., 2010). 150 nM INF1 solution were allowed to infiltrate the intercellular space of *N. benthamiana* leaves using a syringe without a needle (Shibata et al., 2010).

### Virus-Induced Gene Silencing (VIGS)-Mediated Random Gene Silencing

VIGS-mediated random gene silencing was performed as described in Shibata et al. (2016). Briefly, cDNA fragments were prepared using PCR-Select cDNA Subtraction Kit (Clontech, United States). Leaves of *N. benthamiana* were harvested at 1, 3, 6, and 12 h after 150 nM INF1 treatment and used to the prepare tester cDNA. Leaves without treatment were used for the preparation of driver cDNA. BI and HIII library primers (Supplementary Table S1) were used for the second round PCR. Amplified PCR products were digested with *Bam*HI and *Hin*dIII and cloned into the *Bam*HI-*Hin*dIII sites of pTV00 (Ratcliff et al., 2001). Vectors with random cDNA fragments were transformed into *A. tumefaciens* (strain GV3101), and *Agrobacterium*-mediated induction of VIGS was performed as described previously (Shibata et al., 2010) for ˜3,000 individual *Agrobacterium* transformants.

### Construction of Vectors for VIGS, Gene Expression, and Yeast Two-Hybrid Assay

Base vectors, primer sets and templates for the PCR amplification of DNA fragments for vector construction are listed in Supplementary Table S2. Primers used for the construction of vectors are listed in Supplementary Table S1. Gene fragments in pTV00 vectors for VIGS induction were assessed using the SGN VIGS tool (Fernandez-Pozo et al., 2015) to exclude unexpected off-target effects (Supplementary Table S3).

### Virus-Induced Gene Silencing (VIGS), Gene Expression by Agroinfiltration and Stable Transformation of *N. tabacum*

*Agrobacterium*-mediated induction of VIGS and gene expression were performed as previously described (Ratcliff et al., 2001; Shibata et al., 2010). For *P. infestans* inoculation, *N. benthamiana* leaves were inoculated with *A. tumefaciens* (strain GV3101), then downside of detached leaves were inoculated with *P. infestans* 24 h after *Agrobacterium* inoculation. Stable transformants of *N. tabacum* were produced as described previously (Ohshima et al., 1990) using the pEl2Ω vector (Ohtsubo et al., 1999) containing *GFP-NbRan1a* gene under the control of the 35S promoter.

### Quantitative RT-PCR

Total RNAs were isolated from *N. benthamiana* leaves using TRIzol Reagent (Life Technologies, United States) and cDNA synthesis was conducted using ReverTra Ace-α- (Toyobo, Japan) according to the manufacturer’s instructions. Quantitative RT-PCR (qRT-PCR) analysis was performed using LightCycler Quick System 350S (Roche Applied Science, Germany) with Thunderbird SYBR qPCR Mix (Toyobo, Japan). Expression of *N. benthamiana EF-1*α gene was used as an internal standard. Gene-specific primers used for expression analysis were listed in Supplementary Table S4.

### Detection of Poly (A) RNA

Intracellular localization of mRNAs in epidermal and mesophyll cells of *N. benthamiana* was performed as described by Mizuno and Takemoto (2015). Briefly, bleaching and fixation of small pieces of *N. benthamiana* leaves were performed using Fixation cocktail (4x: 240 mM NaCl, 14 mM Na_2_HPO_4_, 6 mM NaH_2_PO_4_, 5.4 mM KCl, 160 mM EGTA), Fixation solution A (1x Fixation cocktail, 2.5% formaldehyde, 5% DMSO, 0.1% Tween20, 50%Heptan), Fixation solution B (1x Fixation cocktail, 5% DMSO, 0.1% Tween20, 50% Heptan), 99.8% Methanol, 99.5% Ethanol, 99.5% Xylen and PerfectHyb plus hybridization buffer (Sigma-Aldrich). Hybridization of probe was performed in PerfectHyb plus hybridization buffer containing 10 nM Alexa Fluor 488-labeled oligo(dT)_45_ (Eurofins Genomics Tokyo, Japan) at 50°C with gentle shaking overnight. Confocal fluorescence images were recorded with a 488 nm excitation source, and Alexa Fluor-488 fluorescence was recorded between 500 and 600 nm using an FV1000-D microscope (Olympus, Japan).

### Capsidiol Extraction and Quantification

Extraction and quantification of capsidiol were performed as previously reported (Matsukawa et al., 2013) with a minor modification. *N. benthamiana* leaves (50 mg) treated with water or 150 nM INF1 were washed in ethyl acetate/cyclohexane (1:1) for 1 h with shaking. Capsidiol extracted in the organic solvent was dried in a 1.5 ml microtube, and dissolved in 150 μl of 67% acetonitrile, and a 5 μl portion was subjected to HPLC analysis under the following conditions. Column: Develosil ODS-UG-3 (4.6 by 150 mm); solvent: 50% acetonitrile (0–3 min), 50–80% acetonitrile (3–13 min), and 80–100% acetonitrile (13–14 min); flow rate: 1.0 ml/min; detection at 205 nm. Capsidiol was eluted at 4.6. The peak areas were used for quantitative analysis.

### Yeast Two-Hybrid and Co-immunoprecipitation Assay

Yeast transformation was performed using *S. cerevisiae* Direct Transformation Kit (Wako, Japan). Yeast two-hybrid assays were performed using MATCHMAKER Two-Hybrid System3 (Clontech) as previously described (Kayano et al., 2018). Briefly, yeast strain AH109 was transformed with prey and bait vectors, pGADT7 and pGBKT7 (Supplementary Table S2) and transformants were isolated on SD medium lacking leucine and tryptophan. Transformants were then cultured on SD medium lacking leucine, tryptophan, histidine and adenine (-LTHA), and growth on the latter indicates an interaction between bait and prey. Co-immunoprecipitation assay was performed as described by Xu et al. (2015) using monoclonal antibodies against GFP (mFX75 012-22541, Wako) and FLAG (anti-DYKDDDDK 018-22381, Wako).

### Fluorescence Microscopy

Fluorescence images were recorded between 425 and 475 nm (calcofluor white), between 495 and 520 nm (GFP) or between 570 and 590 nm (RFP) under a FV1000-D microscope (Olympus) using 405 nm (calcofluor white), 488 nm (GFP), or 559 nm (RFP) excitation.

### Bimolecular Fluorescence Complementation (BiFC) Assay

Bimolecular fluorescence complementation (BiFC) assays was performed as follows. Leaves of *N. benthamiana* were inoculated with *A. tumefaciens* strains containing expression vector pNPP40-nGFP-NbRanBP1-1a and pNPP40-cGFP-Ran1a, or pNPP40-nGFP-NbRan1a and pNPP40-cGFP-RanBP1-1a. Fluorescence images for GFP were recorded ˜2 days after inoculation under a FV1000-D microscope (Olympus) as described above.

### Preparation of Protein Samples and Western Blotting

Preparation of protein samples, quantification of protein concentration and western blot analysis were performed as previously described (Shibata et al., 2010; Ohtsu et al., 2014). Note that RBCL bands were often weaker in *NbRan*BP1*-1*-silenced plants compared with control plants when we applied samples based on their protein concentration determined by Bradford protein assay. Therefore, we adjusted the amount of applied samples for each lane based on the intensity of RBCL bands in Figure 3D.

## Results

### *Nicotiana benthamiana* Ran-Binding Protein 1 Is Required for Resistance to *P. infestans*

**FIGURE 1:**
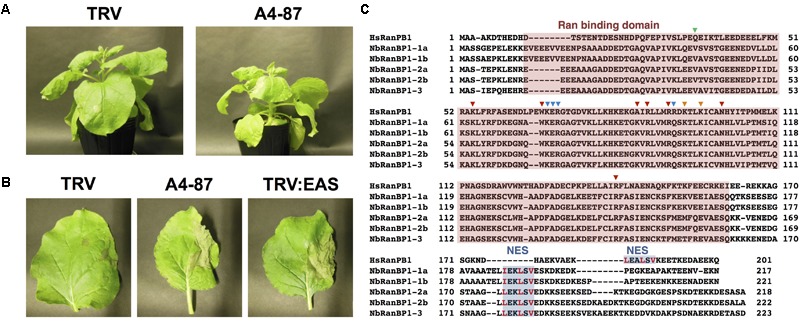
Isolation of NbRanBP1-1 (Ran-binding protein 1-1) as an essential gene of *Nicotiana benthamiana* for resistance to *Phytophthora infestans*. **(A)** Growth phenotype of *N. benthamiana* infected with TRV (control) or gene-silenced line A4-87. Photographs were taken 18 days after the inoculation with A. tumefaciens for VIGS induction. **(B)** Right side of leaves of control (TRV), A4-87 line or NbEAS-silenced *N. benthamiana* were inoculated with *P. infestans*. Photographs were taken 5 days after inoculation. TRV and TRV:EAS infected plants were used as resistant and susceptible control plants, respectively. **(C)** Alignment of the deduced amino acid sequences of Human and *N. benthamiana* RanPB1. Ran binding domain (red) and nuclear export signal, NES (blue) were highlighted. Amino acids for contacts to N-terminus, effector loop and C-terminus of Ran (Vetter et al., 1999) are indicated by green, blue and red arrowheads, respectively. The crucial hydrophobic residues in NES are shown in red letters (Richards et al., 1996).

**FIGURE 2:**
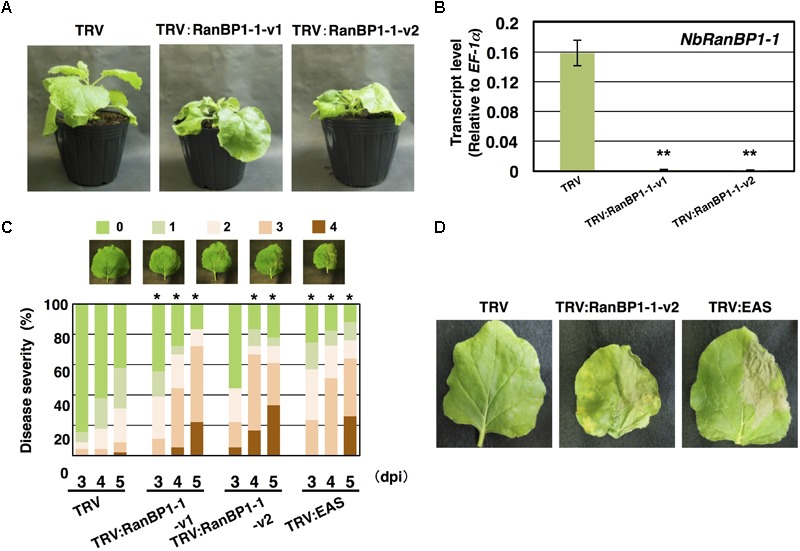
*Nicotiana benthamiana* RanBP1-1 is involved in resistance to *Phytophthora infestans*. **(A)** Growth phenotype of *N. benthamiana* infected with TRV, TRV:NbRanBP1-1-v1 or TRV:RanBP1-1-v2. Photographs of gene-silenced plants were taken 17 days after the inoculation with Agrobacterium tumefaciens for VIGS induction. **(B)** *N. benthamiana* plants were inoculated with TRV, TRV:RanBP1-1-v1 or TRV:RanBP1-1-v2. Expression of NbRanBP1-1 was assessed by qRT-PCR with gene-specific primers. Expression levels of genes were quantified relative to that of constitutively expressing NbEF-1α. Means ± SE (n = 3). Data marked with asterisks are significantly different from control as assessed by the two-tailed student’s t-test: ^∗∗^P < 0.01. **(C)** Right side of leaves of control or gene-silenced plants were inoculated with *P. infestans* and appearance of disease symptoms was categorized into 5 classes according to the severity of disease symptoms. 0, no visible symptom; 1, small wilted spots in inoculated area; 2, browning < 50% of inoculated side of leaf; 3, browning > 50% of inoculated side of leaf; 4, development of disease symptoms over central leaf vein. Plot showing percentage of *N. benthamiana* leaves with disease symptom severities represented in the five classes as shown in the upper panels, for leaves of control, NbRanBP1-1- or NbEAS-silenced plants inoculated with *P. infestans* from 3 to 5 days post-inoculation (dpi). At least 18 leaves from each gene-silenced plants were scored. Data marked with asterisks are significantly different from control as assessed by one-tailed Mann–Whitney U-tests: ^∗^P < 0.05. **(D)** Leaves of control, NbRanBP1-1-silenced or NbEAS-silenced *N. benthamiana* were inoculated with *P. infestans*. Photographs were taken 5 dpi.

Virus-induced gene silencing (VIGS)-based screening has been performed to isolate *N. benthamiana* genes required for resistance to *P. infestans* (Shibata et al., 2016). Using random primers, short cDNA fragments were generated from mRNA of *N. benthamiana* leaves treated with the elicitor INF1, a secretory elicitor protein produced by *P. infestans* (Kamoun et al., 1997), and subsequently inserted into the pTV00 vector (Ratcliff et al., 2001). These vectors were then used to randomly silence *N. benthamiana* genes. The randomly gene-silenced *N. benthamiana* plants (˜3,000 lines) were inoculated with *P. infestans* isolate 08YD1, which is non-pathogenic to mature *N. benthamiana* (Shibata et al., 2011). Development of disease symptoms was assessed for 10 days after inoculation, and 82 plant lines susceptible to *P. infestans* were isolated (Shibata et al., 2016). The cDNA fragments contained in the pTV00 vector used for the production of each of these susceptible lines, were sequenced and listed as candidate genes required for resistance of *N. benthamiana* to *P. infestans*. Further analyses of some isolated genes from this screening have been reported elsewhere (Matsukawa et al., 2013; Ohtsu et al., 2014; Shibata et al., 2016). In this study, we report functional analysis of a gene isolated from line A4-87. A4-87 plants showed minor growth defects and developed disease symptoms by inoculation with *P. infestans* (Figure 1A,B). Disease symptoms of A4-87 were less severe compared with *NbEAS*-silenced plants, in which the expression of the 5-*epi*-aristolochen*e* synthase gene (encoding a key enzyme for capsidiol production) is suppressed (Shibata et al., 2010). The VIGS vector used for the production of line A4-87 contained a 310 bp cDNA fragment. A BLAST search of the cDNA insert sequence in the *N. benthamiana* genome (ver. 1.0.1, Bombarely et al., 2012) revealed that this cDNA fragment is a part of Niben101Scf00439g07002.1 encoding Ran-binding protein 1 (RanBP1) (Figure 1C, Supplementary Figure S1, and Supplementary Table S5). Analysis using the SGN VIGS tool for detection of potential off-target effects (Fernandez-Pozo et al., 2015) identified another gene encoding RanBP1 (Niben101Scf24384g00009.1) as silencing target in A4-87 line. As Niben101Scf00439g07002.1 and Niben101Scf24384g00009.1 are highly homologous, we designated these genes as *NbRanBP1-1a* and *NbRanBP1-1b* (Figure 1C, Supplementary Figure S1 and Supplementary Table S5). Since *N. benthamiana* has an allopolyploid genome (Goodin et al., 2008), it was expected that the two *NbRanBP1-1* genes are derived from different ancestral *Nicotiana* species, and are probably functionally redundant. As *NbRanBP1-1a* and *-1b* genes are highly homologous, we could not design a vector for gene-silencing only a single *NbRanBP1-1* gene. Therefore, *NbRanBP1-1*-silenced plants, with reduced expression of both *NbRanBP1-1a* and *-1b*, are used for following analyses. There are three additional *NbRanBP1* genes which can be identified in the genome of *N. benthamiana* (Supplementary Table S5). Because no homolog of mammalian RanBP2 (also known as Nup358) was identified in plant genomes (Tamura and Hara-Nishimura, 2011; Ohtsu et al., 2014), we avoided using “RanBP2” and designated additional members of *NbRanBP1* homologs in *Nicotiana benthamiana* genome as *NbRanBP1-2a, -2b*, and *-3* (Figure 1C and Supplementary Table S5). NbRanBP1-2 and -3 have similar domain compositions to NbRanBP1-1 including Ran binding motifs and nuclear export signal (NES), but the C-terminal end of the protein was distinguishable from NbRanBP1-1 (Figure 1C). Phylogenetic analysis revealed that Solanaceae RanBP1-1, -2, and -3 proteins are divided in three separate clades (Supplementary Figure S2). *NbRanBP1-2a, -2b*, and *-3* are not detected by the SGN VIGS tool using 310 bp fragment from line A4-87 as query sequence (Supplementary Table S3). To confirm the requirement of *NbRanBP1-1* for disease resistance to *P. infestans*, two VIGS vectors (*NbRanBP1-1*-v1 and *1-1*-v2) were constructed to specifically suppress the expression of *NbRanBP1-1* (Supplementary Figure S1 and Supplementary Table S3). Both *NbRanBP1-1*-silenced *N. benthamiana* lines showed growth defects which are more severe than in the A4-87 line, probably as new vectors have higher efficiency for induction of gene silencing (Figure 1A, 2A), and significantly reduced expression of *NbRanBP1-1* was detected in these gene-silenced plants (Figure 2B). Both *NbRanBP1-1-*silenced plants showed compromised disease resistance to *P. infestans* (Figure 2C,D), confirming that NbRanBP1-1 is required for disease resistance of *N. benthamiana* against *P. infestans*. In the following analyses of this study, VIGS vector *NbRanBP1-1*-v2, which caused consistent growth defects of gene-silenced plants, was chosen for the production of *NbRanBP1-1-*silenced plants. It was also confirmed that expression of *NbRanBP1-2* and *-3* was not reduced in *NbRanBP1-1*-silenced *N. benthamiana* (Supplementary Figure S3). Interestingly, expression of *NbRanBP1-2* was upregulated in *NbRanBP1-1*-silenced *N. benthamiana*, possibly as a feedback of reduced expression of *NbRanBP1-1.*

### NbRanBP1 Is Involved in the Nuclear Export of mRNA and Timely Production of Phytoalexin

**FIGURE 3:**
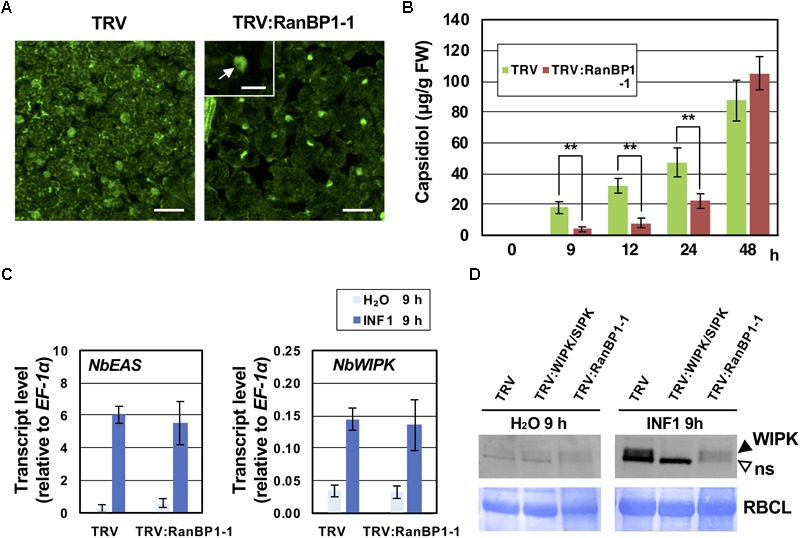
NbRanBP1 is involved in efficient phytoalexin production. **(A)** Distribution of poly(A) RNA in mesophyll cells of control (TRV) and NbRanBP1-1-silenced *Nicotiana benthamiana* leaves probed with oligo(dT)_45_ labeled with Alexa 488. Arrow in inset indicates a nucleolus. Bars = 50 μm for main panels and 20 μm for inset. **(B)** Capsidiol was extracted from control (TRV), or NbRanBP1-1-silenced *N. benthamiana* at indicated time after 150 nM INF1 treatment and quantified by HPLC. Data are means ± SE (n = 3–27). Note that no production of capsidiol was detected for water-treated samples. Data marked with asterisks are significantly different from control as assessed by the two-tailed student’s t-test: ^∗∗^P < 0.01. **(C)** Leaves of control or NbRanBP1-1-silenced plants were treated with 150 nM INF1 and the expression of NbEAS and NbWIPK was quantified relative to that of constitutively expressing NbEF-1α 9 h after the treatment. Data are means ± SE (n = 3). **(D)** Control or gene-silenced *N. benthamiana* leaves were treated with water or 150 nM INF1 and harvested 9 h after treatment. Total proteins were extracted from leaves and NbWIPK was detected using Anti-NtWIPK antibody. Bands for the ribulose-1,5-bisphosphate carboxylase large subunit (RBCL) were monitored by CBB staining (see Experimental procedures). Results shown are representative of at least 3 separate experiments. Open arrowhead indicates the non-specific (ns) band detected by Anti-NtWIPK antibody (Seo et al., 2007).

We have previously identified *NbNup75*, encoding a component of NPC, as an essential gene for the resistance of *N. benthamiana* to *P. infestans*. *NbNup75*-silenced *N. benthamiana* showed abnormal accumulation of mRNA in nuclei, and caused reduced production of capsidiol, a sesquiterpenoid phytoalexin produced by *Nicotiana* species during the induction of disease resistance (Ohtsu et al., 2014). Given that RanBP1 is a conserved protein among eukaryotic organisms serving as a regulator of nucleo-cytoplasmic transport of proteins and RNAs (Kehlenbach et al., 1999), we expected that functional defects similar to *NbNup75*-silenced plants would be observed in *NbRanBP1-1*-silenced *N. benthamiana*. To examine the effect of *NbRanBP1-1* silencing on nucleo-cytoplasmic transport of mRNA, the subcellular distribution of mRNA was assessed using Poly (A) RNA *in situ* hybridization. In control plants, Alexa Fluor 488-labeled (AF488) Oligo (dT)_45_ detected mRNA in cytoplasm and nuclei, whereas stronger fluorescence signal at nuclei was observed in *NbRanBP1*-silenced plants same as in *NbNup75*-silenced plants (Figure 3A; Ohtsu et al., 2014). This result suggested that *NbRanBP1-1*-silenced plants have defects in the nuclear export of mRNA, which is consistent with a previous report showing that mutation in yeast *RanBP1* (*YRB1*) caused reduced poly(A)^+^ RNA export (Künzler et al., 2001). To analyze the effect of *NbRanBP1-1* silencing on phytoalexin production, accumulation profiles of capsidiol were compared between control and *NbRanBP1-1*-silenced *N. benthamiana* leaves after treatment with INF1 elicitor (Figure 3B). Capsidiol production was significantly lower in *NbRanBP1-1*-silenced plants compared with control plants from 9 to 24 h after INF1 treatment, however, similar amounts of capsidiol had accumulated 48 h after INF1 treatment (Figure 3B). In contrast, expression of *NbEAS*, which encodes the key enzyme for capsidiol production, was not affected by gene silencing of *NbRanBP1-1* at 9 h after INF1 treatment (Figure 3C). These results indicate that NbRanBP1-1 is not required for transcriptional up-regulation of *NbEAS*, but is involved in some process following the transcription of the defense gene, enabling quick production of phytoalexins during the induction of disease resistance.

### NbRanBP1 Is Required for Translation of NbWIPK, but Not the Expression of the NbWIPK Gene, in the Early Stage of Defense Induction

The MAP kinases, NbWIPK and NbSIPK, are central signaling factors for the induction of defense responses in *N. benthamiana* (Zhang and Klessig, 1998; Romeis et al., 1999; Ishihama et al., 2011). We previously reported that the NbWIPK/NbSIPK-mediated up-regulation of ethylene production is essential for the enhanced expression of genes for capsidiol production such as *NbEAS* and *NbEAH* (Shibata et al., 2010; Ohtsu et al., 2014). Transcription of *NbWIPK* is enhanced by INF1 treatment, while expression of NbSIPK is essentially constitutive (Ohtsu et al., 2014). As production of capsidiol is significantly reduced at 9 h after INF1 treatment in *NbRanBP1-1*-silenced plants (Figure 3B), we examined the expression of *NbWIPK* genes in *NbRanBP1-1*- silenced plants. The *NbWIPK* gene was up-regulated at 9 h after INF1 treatment in control plants and gene silencing of *NbRanBP1-1* had no significant effect on the expression of *NbWIPK* (Figure 3C). Accumulation of NbWIPK proteins was also examined using an Anti-NtWIPK antibody (Seo et al., 1999). Gene silencing of *NbRanBP1-1* caused a significant reduction of NbWIPK protein in INF1 treated leaves (Figure 3D). A non-specific band (ns in Figure 3D) detected by the Anti-NtWIPK antibody was also reduced in *NbRanBP1-1*-silenced plants. These results indicated that NbRanBP1-1 is not required for transcriptional up-regulation of defense genes, but is essential for the production of defense-related proteins during the induction of disease resistance.

### NbRanBP1 Interact With NbRan Around the Nuclear Rim and Cytosol in Epidermal Cells of *N. benthamiana*

**FIGURE 4:**
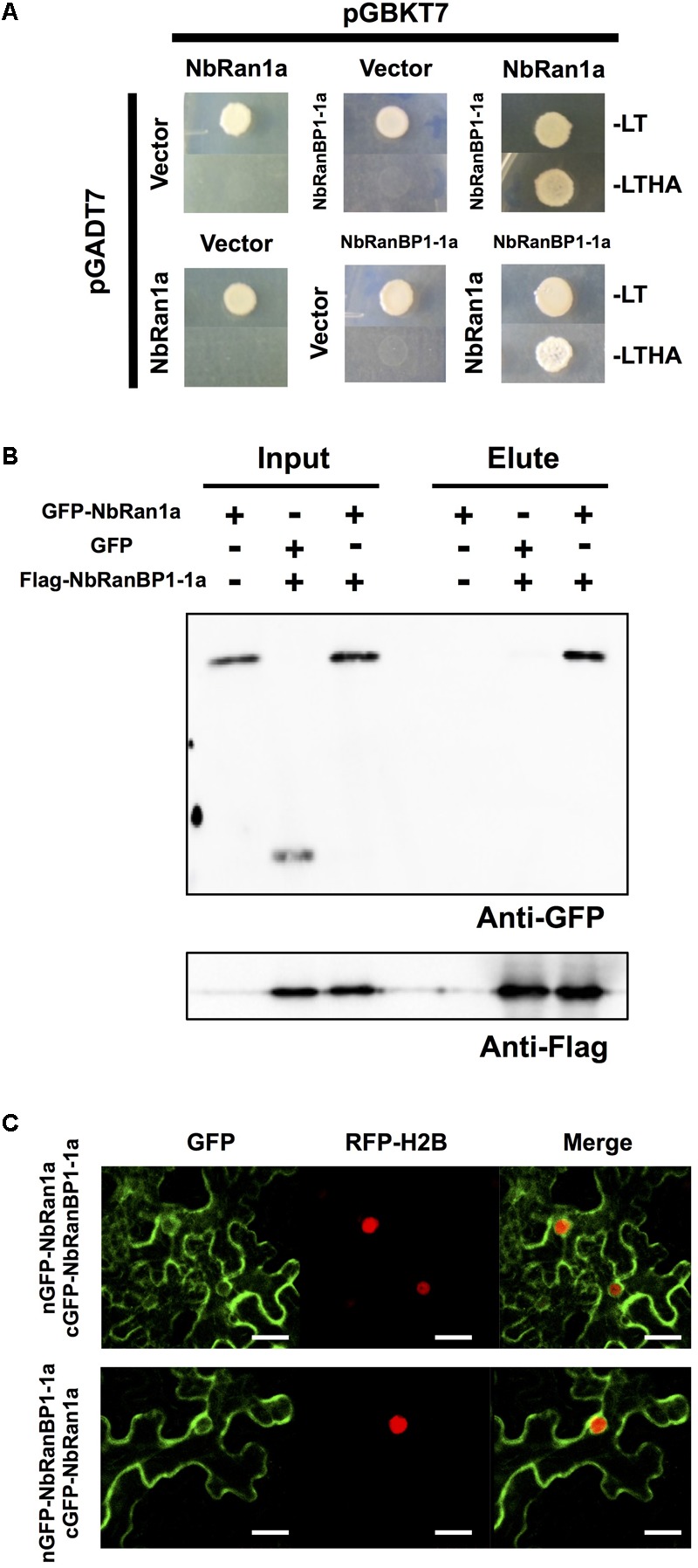
NbRan1a interacts with NbRanBP1-1a. **(A)** Yeast two-hybrid assays of the interactions between NbRan1a and NbRanBP1-1a. Yeast strain AH109 was transformed with prey and bait vector, pGADT7 and pGBKT7, as indicated and plated on SD medium lacking leucine and tryptophan (-LT) or lacking leucine, tryptophan, histidine and adenine (-LTHA). Growth on the latter indicates an interaction between bait and prey. **(B)** Leaves of *Nicotiana benthamiana* were inoculated with Agrobacterium tumefaciens containing expression vectors for either GFP, GFP-NbRan1a or FLAG-NbRanBP1-1a. Leaves were harvested 48 h after inoculation. Co-immunoprecipitation was carried out with anti-FLAG peptide, and proteins were detected by immunoblot with anti-FLAG antibody or anti-GFP antibody. **(C)** Bimolecular fluorescence complementation (BiFC) assays showing interactions between NbRan1a and NbRanBP1-1a. *N. benthamiana* leaves were inoculated with A. tumefaciens strains for expression of nGFP-NbRanBP1-1a and cGFP-Ran1a (top) or nGFP-NbRan1a and cGFP-RanBP1-1a (bottom). Histone H2B tagged with TagRFP was co-expressed as the nuclear marker protein. Bars = 30 μm. nGFP and cGFP are N- (2-174) and C- (175-239) terminal portion of GFP, respectively.

In eukaryotic organisms, the small GTP-binding protein Ran is a key regulator of transports across the nuclear envelope. The direction of transport is principally controlled by asymmetric distribution of Ran in nuclei (GTP-bound form) and in cytosol (GDP-bound form) (Melchior, 2001). In yeast and mammalian cells, RanBP1 directly interacts with Ran to promote transformation of Ran from GTP-bound to GDP-bound form, followed by the release of cargo into the cytosol (Ren et al., 1995; Yokoyama et al., 1995). To examine the interaction between *N. benthamiana* RanBP1-1 and Ran, we first identified Ran genes in the genome of *N. benthamiana*. Four *Ran* genes were identified in the *N. benthamiana* genome and designated *NbRan1a, NbRan1b, NbRan2*, and *NbRan3* (Supplementary Figure S4A and Supplementary Table S6). Comparison of deduced amino acid sequences of NbRan revealed that they are highly conserved, though NbRan3 has an incomplete G3 domain (Supplementary Figure S4A). Expression levels of *NbRan1* and *NbRan2* genes in *N. benthamiana* leaves were not significantly affected by treatment with INF1 elicitor, while expression of *NbRan3*, at least in leaf tissue, was not detected (Supplementary Figure S4B), thus it may be possible that NbRan3 is a pseudogene. To detect the interaction between NbRanBP1-1 and NbRan1, two-hybrid and immuno-precipitation assays were performed. Genes for *NbRanBP1-1a* and *NbRan1a* were introduced into the bait and prey vectors respectively, and transformed into yeast. Only yeast cells expressing both NbRanBP1-1a and NbRan1a grew in histidine and adenine deficient SD media, indicating interaction between NbRanBP1-1a and NbRan1a (Figure 4A). To detect the interaction between NbRanBP1-1a and NbRan1a in plant cells, GFP-tagged NbRan1a and flag-tagged RanBP1-1a were co-expressed in *N. benthamiana* leaves by agroinfiltration, and co-immunoprecipitation assay was performed using anti-GFP antibody (Figure 4B). Flag-tagged NbRanBP1-1a was co-precipitated with GFP-NbRan1a but not with GFP, which further confirmed that NbRanBP1-1a can interact with Ran1a (Figure 4B). As the size of the flag-tagged NbRanBP1-1a was larger than predicted by the amino acid sequence, mass analysis was performed and confirmed that the protein precipitated by Anti-flag antibody is, in fact, NbRanBP1-1a. Detection of RanBP1 bands with larger than expected molecular weight in SDS-PAGE gel was also reported for human RanBP1 (Bischoff et al., 1995).

Bimolecular fluorescence complementation (BiFC) assay was performed to detect subcellular localization of interaction between NbRanBP1-1a and NbRan1a. GFP fluorescence was detected by *Agrobacterium*-mediated transient expression of NbRanBP1-1a tagged with the C-terminal portion of GFP (cGFP-NbRanBP1-1a) and NbRan1a tagged with the N-terminal portion of GFP (nGFP-NbRan1a), or co-expression of nGFP-NbRanBP1-1a and cGFP-NbRan1a, confirmed the interaction between NbRan1a and NbRanBP1-1a in *N. benthamiana* cells. GFP fluorescence was detected on the outer side of the nuclear envelope as well as the cytosol and cytosolic strands of epidermal cells of *N. benthamiana* (Figure 4C).

### Gene Silencing of *NbRan* Compromised Resistance to *P. infestans* and INF1-Induced Phytoalexin Production

**FIGURE 5:**
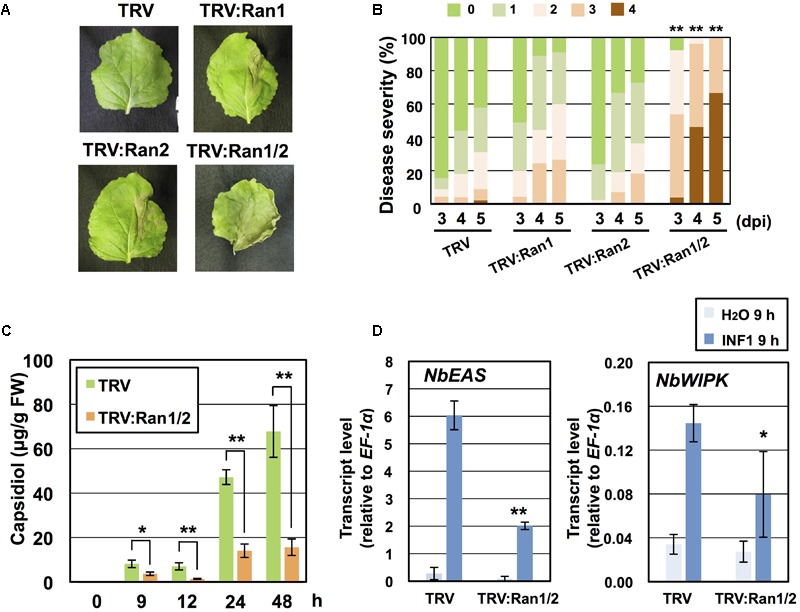
NbRan is essential for the resistance of *Nicotiana benthamiana* to *Phytophthora infestans*. **(A)** Leaves of control (TRV), NbRan1-, NbRan2-, NbRan1/NbRan2-silenced *N. benthamiana* were inoculated with *P. infestans*. Photographs were taken 5 days post-inoculation (dpi). **(B)** Pathogen resistance was categorized into 5 classes according to the severity of disease symptoms as described in Figure 2. Plot showing percentage of *N. benthamiana* leaves with disease symptom severities represented in the five classes, for leaves of control, NbRan1-, NbRan2-, NbRan1/2-silenced plants inoculated with *P. infestans* from 3 to 5 dpi. At least 38 leaves from each gene-silenced plants were scored. Data marked with asterisks are significantly different from control as assessed by one-tailed Mann–Whitney U-tests: ^∗∗^P < 0.05. **(C)** Capsidiol was extracted from control (TRV), or NbRan1/NbRan2-silenced *N. benthamiana* at 0, 9, 12, 24, and 48 h after treatment with 150 nM INF1 and quantified by HPLC. Data are means ± SE (n = 6–15). Note that no production of capsidiol was detected for water-treated samples. **(D)** Leaves of control (TRV) or NbRan1/2-silenced plants were treated with 150 nM INF1 and the expression of NbEAS and NbWIPK was quantified relative to that of constitutively expressing NbEF-1α 9 h after the treatment. Data are means ± SE (n = 3). Data marked with asterisks are significantly different from control as assessed by the two-tailed student’s t-test: ^∗^P < 0.05, ^∗∗^P < 0.01.

Requirement of NbRan in resistance of *N. benthamiana* to *P. infestans* was investigated. As we hardly detected the expression of *NbRan3* in leaf tissue of *N. benthamiana* (Supplementary Figure S4B), we constructed VIGS vectors for silencing of *NbRan1, NbRan2* or double silencing of *NbRan1* and *NbRan2* (Supplementary Figures S5A,B). Growth of *NbRan1-* and *NbRan2*-silenced plants was comparable to control plants, whereas *NbRan1*/*NbRan2* double*-*silenced plants showed a dwarf phenotype, similar to *NbRanBP1-1*-silenced plants (Figure 2A and Supplementary Figure S5C). Same as *NbRanBP1-1*-silenced plants, preferential nuclear accumulation of mRNA was observed in *NbRan1/NbRan2-*silenced plants (Supplementary Figure S5D). Development of disease symptoms of control and *NbRan*-silenced plants was compared from 3 to 5 days post-inoculation with *P. infestans*. Although *NbRan1-* and *NbRan2-*silenced plants tended to show slightly more severe disease symptoms compared with control plants, no statistically significant decrease of *P. infestans* resistance was observed by silencing of a single *NbRan* gene. In contrast, *NbRan1*/*NbRan2*-silenced plants showed obvious severe disease symptoms (Figure 5A,B). Production of capsidiol in control and *NbRan1/NbRan2-*silenced plants was quantified in *N. benthamiana* leaves treated with INF1. *NbRan1/NbRan2-*silenced plants showed reduced production of capsidiol from 9 to 48 h after INF1 treatment (Figure 5C). In contrast with *NbRanBP1-1*-silenced plants, expression of *NbEAS* and NbWIPK was reduced 9 h after INF1 treatment (Figure 5D), indicating that NbRan1 and NbRan2, but not NbRanBP1-1, are involved in transcriptional up-regulation of defense genes. In case of *NbRan1-* or *NbRan2-*silenced plants, production of capsidiol was not significantly affected at 24 h after INF1 treatment, while reduction of capsidiol production was obvious at 48 h after treatment for both *NbRan-*silenced plants (Supplementary Figure S6A). mRNA tended to accumulate in nuclei of *NbRan1*- and *NbRan2-*silenced plants (Supplementary Figure S6B). These results indicate that NbRan1 and NbRan2 have overlapping functions concerning nuclear export of mRNA and production of capsidiol during the induction of disease resistance in *N. benthamiana*.

### Nuclear Accumulation of NbRan1 Is Reduced During the Induction of Disease Resistance

**FIGURE 6:**
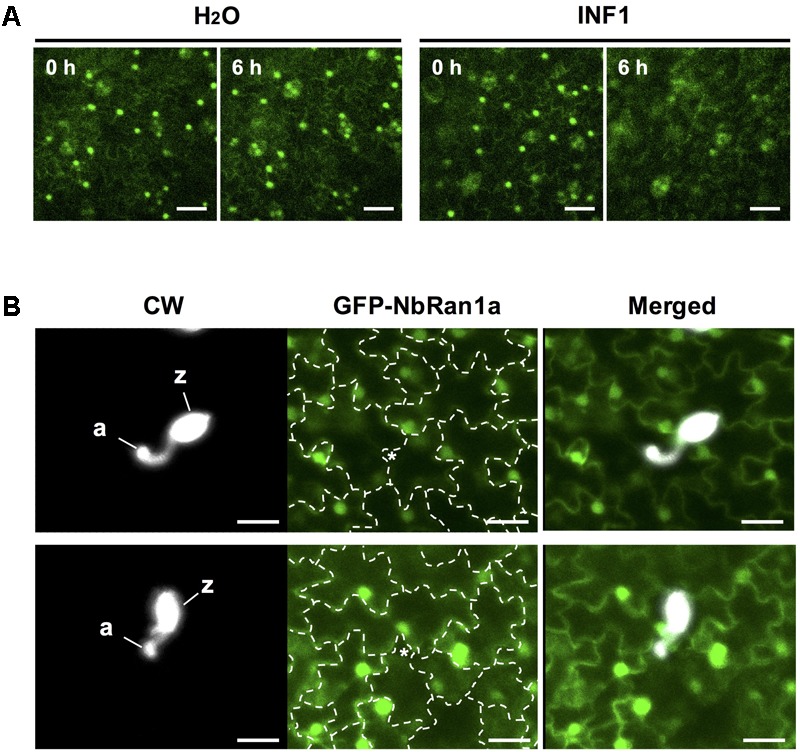
Nuclear accumulation of NbRan1 is reduced during the induction of disease resistance. **(A)** Leaves of transgenic Nicotiana tabacum expressing GFP-NbRan1a was treated with water or 150 nM INF1. GFP fluorescence was monitored by confocal laser scanning microscopy at 0 or 6 h after the treatment. Representative images of 5 separate time course scannings. Bar = 50 μm. **(B)** Leaves of transgenic N. tabacum expressing GFP-NbRan1a were inoculated with *Phytophthora infestans* and GFP fluorescence was monitored by confocal laser scanning microscopy at 12 h after the inoculation. *P. infestans* was visualized by calcofluor white (CW) staining. Representative images of at least 10 infection sites observed. The junctions of epidermal cells are outlined by dashed lines and penetration sites are indicated by asterisks. a, appressorium-like swelling; z, zoosporangium. Bar = 30 μm.

Transgenic *N. tabacum* expressing GFP-NbRan1a was produced to monitor the intracellular distribution of NbRan1a during the induction of disease resistance. As previously observed for mammalian Ran, GFP-NbRan1a predominantly localized to the nuclei with low levels of cytoplasmic fluorescence (Hutchins et al., 2009; Figure 6A). Upon treatment with INF1, nuclear accumulation of GFP-NbRan1a was reduced and diffuse GFP fluorescence was observed in the cytosol from 3 to 6 h after the treatment (Figure 6A). Attempts of penetration by *P. infestans* also caused a loss of nuclear accumulation of GFP-NbRan1a. Approximatey 12 h after inoculation with *P. infestans*, epidermal cells which had been directly targeted by *P. infestans* showed greatly reduced GFP fluorescence in nuclei, and nuclei of surrounding cells tended to have migrated closer to the penetrated cells, indicating a reaction of surrounding cells to attacked cells (Figure 6B). These observations suggest that nuclear accumulation of NbRan1a is reduced during the induction of disease resistance. As gene silencing is not applicable to transgenic *N. tabacum* used above, *N. benthamiana* was used in following experiments.

### Gene-Silencing of *NbRanBP1-1* Affects Nuclear-Cytoplasmic Distribution of NbRan

**FIGURE 7:**
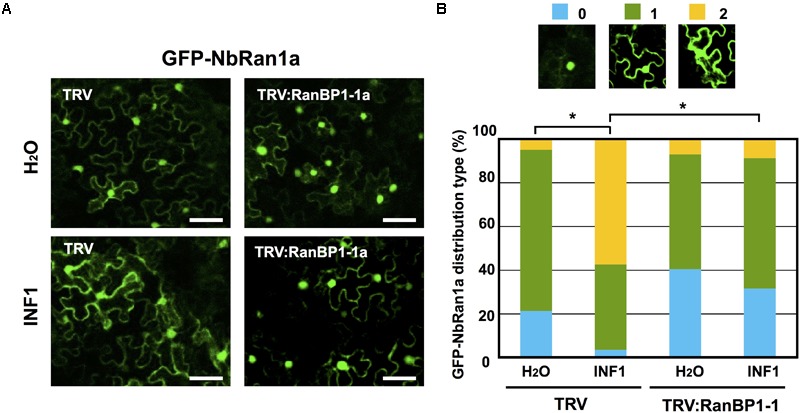
Gene-silencing of NbRanBP1-1 affects nuclear-cytoplasmic distribution of NbRan during the induction of disease resistance. **(A)** Leaves of control (TRV) or NbRanBP1-silenced *Nicotiana benthamiana* were inoculated with Agrobacterium tumefaciens containing expression vector for GFP-NbRan1a. Leaves expressing GFP-Ran1a were treated with water or 150 nM INF1, and GFP fluorescence was monitored by confocal laser scanning microscopy 12 h after treatment. Bars = 50 μm. **(B)** Distribution of GFP-NbRan1a in the epidermal cell was categorized into 3 classes. 0, nuclear localization; 1, nuclear and cytoplasmic localization; 2, nuclear and intense cytoplasmic localization with visible cytoplasmic strands. Plot showing percentage of *N. benthamiana* cells with distribution of GFP-NbRan1a represented in the three classes as shown in upper panels, for control (TRV) or NbRanBP1-1-silenced plants treated with water or INF1 at 12 h after treatment. At least 190 cells from control or silenced plants were scored for each treatment. Data marked with asterisks are significantly different as assessed by one-tailed Mann–Whitney U-tests: ^∗^P < 0.05.

To investigate the role of NbRanBP1-1 in nuclear-cytoplasmic distribution of NbRan, GFP-NbRan1a was expressed in leaves of control or *NbRanBP1-1*-silenced *N. benthamiana* by *Agrobacterium*-mediated transient expression. As in the case of transgenic *N. tabacum*, the majority of signal of GFP-NbRan1a was detected in the nuclei, but cytosolic GFP signal was higher than in stable *N. tabacum* transformants (Figure 7A). After INF1 treatment, an increase of cytosolic GFP-NbRan1a was detected, suggesting that induction of plant defense can stimulate the export of Ran from nucleoplasm to cytosol (Figure 7A,B). Leaves transiently expressing NbRan1a were also inoculated with *P. infestans*. In this experiment, histone-2B-RFP (H2B-RFP) was co-expressed with GFP-NbRan1a in *N. benthamiana* leaves to ensure that nuclei were not collapsed at the time of observation. In cells directly attacked by *P. infestans*, reduction of nuclear GFP-NbRan1a was observed, but nuclear accumulation of H2B-RFP was not affected (Supplementary Figure S7), which indicated the nuclear export of Ran during the attack of *P. infestans*.

Subcellular localization of GFP-NbRan1a in control and *NbRanBP1-1*-silenced plants was compared. Although the distribution of GFP-NbRan1a was not significantly affected by gene-silencing of *NbRanBP1-1* in water treated cells, GFP-NbRan1a preferentially localized to the nuclei in *NbRanBP1-1*-silenced cells even after treatment of INF1 (Figure 7A,B). These results show that NbRanBP1-1 is involved in efficient nuclear export of NbRan1a.

### Nuclear Pore Proteins, NbNup75 and NbNup160 Are Involved in INF1-Induced Nuclear Export of NbRan1a

In this study, *NbNup160*-silenced plants were also produced to assess the role of the Nup107-160 subcomplex (Supplementary Figure S8). Arabidopsis Nup160 has been reported to be required for disease resistance and normal subcellular localization of mRNA (Dong et al., 2006; Wiermer et al., 2012). The vertical growth rate of *NbNup160*-silenced plants is almost as normal as wild type, though their leaves tend to be narrower than wild type (Supplementary Figure S8A). *NbNup160*-silenced plants showed more severe disease symptoms compared with control plants after *P. infestans* inoculation (Supplementary Figures S8C,D). Enhanced mRNA accumulation in nuclei was observed in *NbNup160*-silenced plants, comparable to *NbNup75-*silenced plants (Supplementary Figure S9A; Ohtsu et al., 2014). Reduced accumulation of capsidiol was observed in *NbNup160*-silenced plants as well (Supplementary Figure S9B), suggesting that NbNup160 is involved in the similar process to *NbNup75-* and *NbRanBP1-1* for the induction of disease resistance. Nucleo-cytoplasmic distribution of NbRan1a was investigated in *NbNup-*silenced plants. Same as in the case of *NbRanBP1*-*1-*silenced plants, gene silencing of either *NbNup75* or *NbNup160* caused lower fluorescence levels of GFP-NbRan1 in the cytosol after INF1 treatment (Supplementary Figure S10), thus suggesting that NbNup75 and NbNup160 are involved in INF1-induced nuclear export of NbRan1a.

## Discussion

**FIGURE 8:**
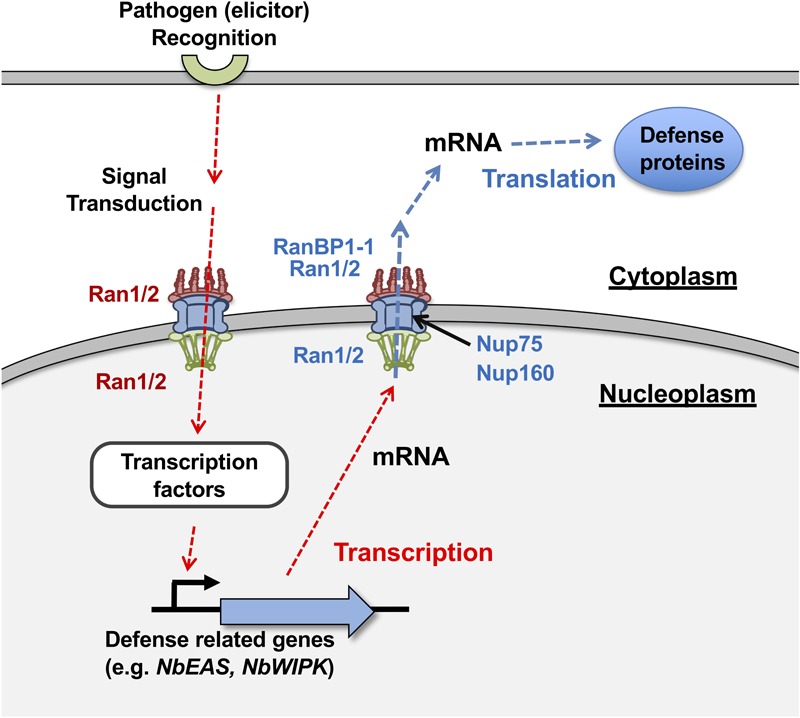
A hypothetic model for the role of NbRanBP1-1 and NbRan in disease resistance of *Nicotiana benthamiana* against *Phytophthora infestans*. (Red dotted arrows) Recognition of pathogen leads to the transcriptional up-regulation of defense-related genes (i.e., NbEAS and NbWIPK) in early stage of defense response. Note that this process was affected by the silencing of NbRan1/2 but not by NbRanBP1-1. (Blue dotted arrows) mRNAs of induced genes are translocated to cytoplasm for the translation of gene products. Both NbRan1/2 and NbRanBP1-1 are involved in this process.

Upon the attempted infection of pathogens, plant cells recognize the structure or activity of pathogen-derived molecules, then transduce the signal into nuclei to enhance the expression of large numbers of defense-related genes. After mRNAs of defense-related genes are transcribed in the nucleus, they are exported to the cytoplasm through nuclear pores where the translation of proteins occurs (Figure 8). The export of increased mRNA to cytosol could potentially be a limiting factor for timely activation of plant defense, however, regulation of nuclear export of mRNA in plant cells during stress responses is poorly understood. Ran GTPase cycle is known as one of the major nucleo-cytoplasmic transport mechanisms of RNAs and proteins in eukaryotic cells. In this study, we isolated a *N. benthamiana* gene related to nucleo-cytoplasmic transport, NbRanBP1-1, as an essential factor for appropriate induction of plant defense against *P. infestans*. Silencing of NbRanBP1-1 compromised translation of defense-related MAP kinase, NbWIPK, but the expression of NbWIPK gene was not significantly affected by the silencing. Therefore, NbRanBP1 is probably involved in the export of mRNA, which is critical for timely induction of effective plant defense against pathogen infection (Figure 8).

### NbRanBP1-1 and NbRan1/2 Are Involved in Nuclear Export of mRNA

Previously, we have reported that *NbNup75-*silenced plants show abnormal accumulation of mRNA in the nucleus (Ohtsu et al., 2014). *NbNup160-*silenced plants also showed a similar pattern of mRNA accumulation (Supplementary Figure S9), confirming the role of Nup107-160 subcomplex in nuclear export of mRNA. Involvement of Arabidopsis and mammalian Nup107-160 subcomplex and yeast Nup84 subcomplex in nuclear export of mRNA also have been reported (Doye and Hurt, 1997; Wiermer et al., 2012). In this study, we detected accumulation of mRNA in *NbRanBP1*- and *NbRan1/2-*silenced plants. Efficient transport of general mRNA was blocked in *NbRanBP1-1*-silenced plants as abnormal accumulation of mRNA was observed in normal growth condition. The accumulation of mRNA in nuclei *per se* has no major impact for their normal growth as no obvious growth defect was observed in *NbNup75*-silenced *N. benthamiana* and Arabidopsis *nup75* mutants, which also caused nuclear accumulation of mRNA (Wiermer et al., 2012; Ohtsu et al., 2014). Delayed induction of capsidiol production in *NbRanBP1-1*-silenced plants probably indicated that efficient export of transiently increased mRNA is required during the induction of stress responses. In fact, Arabidopsis *nup160* mutant showed reduced cold tolerance and disease resistance (Dong et al., 2006; Wiermer et al., 2012).

Although a previous report indicated that mutation of yeast *RanBP1* (*YRB1*) caused reduced nuclear export of poly(A)^+^ RNAs (Künzler et al., 2001), it is generally known that the majority of mRNA is exported via a mechanism mediated by a heterodimer of Mex67 (Nxf1 in mammal) and Mtr2 (Nxt1 in mammal) via TREX (transcription-export) complex, which is not relying on the Ran GTP cycle (Santos-Rosa et al., 1998; Carmody and Wente, 2009). In Arabidopsis, a component of TREX complex, HPR1, was shown to be required for mRNA export and resistance to bacterial and oomycete pathogens (Pan et al., 2012). A subset of mRNAs was known to be exported in a Ran-dependent manner. A nuclear export receptor, Crm1 in the mammal (Xpo1 in yeast), is involved in Ran-dependent nuclear export of mRNAs. Cargo mRNAs exported via the Ran/Crm1 dependent mechanism includes transcripts of the so-called early response genes (ERGs), such as *Interferon alpha-1 IFNA1* and the transcription factor *c-fos* (Brennan et al., 2000; Kimura et al., 2004). Analogous to mammalian research, nuclear export of a portion of mRNAs for initial induction of plant disease resistance might be under the control of a Ran-mediated mechanism.

### NbRan1/2, but Not NbRanBP1-1, Is Involved in the Initial Signal Transduction for Up-Regulating the Expression of Defense Genes

The small GTPase Ran is the central regulator of nucleo-cytoplasmic transport, conserved among yeast, vertebrates, and plants (Melchior, 2001). Ran is involved in both import and export of cargos transported across the nuclear envelope. The GDP-bound form of Ran (Ran-GDP) is generally localized in the cytoplasm, and forms a complex with nuclear transport receptors (i.e., importins), as well as the cargo which is to be imported into the nucleus. For nuclear export of cargos, transport receptors (i.e., exportins) bind their cargo and Ran-GTP. Ran GTPase-activating protein (Ran GAP) and RanBP1 are localized on the cytoplasmic side of the NPC and can proceed dissociation of the cargo from the complex as well as activating GTP hydrolysis of Ran. Although such general roles of Ran for nucleo-cytoplasmic transport have been well documented in yeast and vertebrate systems, mechanism for selective transport of cargos in particular condition such as stress response is less understood.

Silencing of *RanBP1-1* had no obvious effect on transcriptional up-regulation of *NbEAS* and *NbWIPK* genes during the induction of disease resistance, whereas expression of defense genes was significantly reduced in *NbRan1/2*-silenced plants (Figure 3C, 5D). These results suggest that NbRan1/2, but not NbRanBP1-1, is required for nuclear import of signaling factor(s) for induction of defense-related genes. This hypothesis is consistent with the known role of Ran in both import and export, while RanBP1 is involved in the export of cargos (Figure 8).

In *NbRanBP1-1*-silenced plants, accumulation of phytoalexin was reduced from 9 to 24 h after treatment with INF1, but it was comparable with control plants at 48 h after treatment (Figure 3B). *NbRanBP1-1-*silenced plants showed reduced resistance to *P. infestans*, indicating that timely production of capsidiol is essential for plant resistance. Mammalian RanBP1 does not directly activate the GTPase of Ran but enhances hydrolysis of Ran-GTP by RanGAP (Bischoff et al., 1995). Therefore, gene silencing of *RanBP1-1* may cause delayed hydrolysis of Ran-GTP, resulting in slower export of cargos which are required for the production of capsidiol.

### NbRanBP1-1, NbNup75, and Nb160 Are Involved in the Defense-Induced Nuclear Export of NbRan1

In transgenic *N. tabacum* expressing GFP-NbRan1a, nuclear accumulation of GFP-NbRan1a was reduced after INF1 treatment, as well as in the cells directly attacked by *P. infestans* (Figure 6). A marked increase of GFP signal in the cytosol after INF1 treatment or pathogen penetration was observed in *N. benthamiana* leaf cells expressing GFP-NbRan1a which was introduced via Agroinfiltration (Figure 7 and Supplementary Figure S7). These results suggested that elicitor treatment or pathogen attack can alter the distribution of NbRan1a, possibly via enhanced nuclear export of NbRan1a. In human cells, osmotic stress can decrease the level of Ran in the nucleus, while cytosolic Ran is increased (Kelley and Paschal, 2007). Treatment of H_2_O_2_, heat shock and UV irradiation also enhanced the ratio of cytosolic Ran in HeLa cells, indicating that various stresses can affect the intracellular localization of Ran as well (Yasuda et al., 2006). Changes in the nuclear/cytoplasmic distribution of Ran during plant defense could be an important process for changing the directionality of transports across the nuclear envelope. Gene silencing of either NbRanBP1, NbNup75 or NbNup160 inhibited this process, which may be the determining cause for the decreased resistance of these gene-silenced plants against *P. infestans*.

### NbRanBP1 and NbRan1/2 Are Required for Disease Resistance as Well as Normal Plant Growth of *N. benthamiana*

*NbRanBP1-1-* and *NbRan1/2-*silenced plants showed a dwarf phenotype as well as reduced resistance to *P. infestans*. It should be noted that dwarfing is not necessarily the direct cause of decreased disease resistance, since we have isolated gene-silenced *N. benthamiana* lines which showed dwarfing and normal or even enhanced disease resistance during our VIGS-based screening. Silencing of *NbNup75* and *NbNup160*, in contrast, had no or only a minor effect on the growth of *N. benthamiana*, despite the fact that they showed abnormal mRNA accumulation in nuclei same as *NbRanBP1-1-* and *NbRan1/2-*silenced plants (Ohtsu et al., 2014; Figure 3A and Supplementary Figures S5D, S9A). Yeast Nup85 (Nup75) and Nup160 are components of the Y-shaped heptameric complex which assembles to form the outer rings of the NPC (Kampmann and Blobel, 2009). Given that the NPC is a fundamental machinery for all eukaryotic cells, it was unexpected that silencing of genes for such a seemingly essential component of the nuclear pore scaffold showed only a minor effect on growth. Similarly, in *L. japonicus*, mutations in *Nup85* (*Nup75*), *Nup133*, and *Seh1* showed defects in symbiotic interactions with rhizobia and mycorrhizal fungi, but plant growth was not significantly affected (Kanamori et al., 2006; Saito et al., 2007; Groth et al., 2010). Detailed microscopic observation revealed that *L. japonicus* mutants lacking either *nup85* or *nup133* did not exhibit severe alterations in NPC distribution or nuclear envelope structure, but *nup85*/*nup133* double mutants showed severe growth defects, which suggested a certain level of redundancy in the Nup components for NPC function (Binder and Parniske, 2014). In yeast, growth defects of *nup85* mutants are less pronounced at lower temperatures, thus loss of a Nup component would cause only a quantitative change in NPC transport capabilities which appears to have a minor effect under normal growth conditions.

While double silencing of *NbRan1/2* caused growth defects, specific silencing of either *NbRan1* or *NbRan2* showed normal growth (Supplementary Figure S5B). Decreased production of capsidiol was detected for both *NbRan1*- and *NbRan2*-silenced plants at 48 h, but not at 24 h after INF1 treatment (Supplementary Figure S6A), and resistance of *NbRan1*- and *NbRan2*-silenced plants to *P. infestans* was not significantly affected (Figure 5B). In *NbRan1/2-*silenced plants, production of capsidiol was significantly reduced from 9 to 48 h after INF1 treatment, and resistance to *P. infestans* was compromised (Figure 5B,C). These results indicate that Ran1 and Ran2 have redundant function in growth as well as in the induction of phytoalexin production. Although not statistically significant, *NbRan1*-silenced plants tend to be weaker to *P. infestans* (Figure 5B) and produce lower amounts of capsidiol (Supplementary Figure S6A) compared with *NbRan2*-silenced plants, perhaps because of the higher expression level of *NbRan1* under physiological conditions (Supplementary Figure S4B).

NbRanBP1-1 and the other two members of NbRanBP1, NbRanBP1-2, and 1-3, probably have distinct functions. Transgenic Arabidopsis lines expressing antisense *RanBP1c* became hypersensitive to auxin, which resulted in a long-rooted phenotype with less lateral roots, but growth defects of above ground tissues were not reported (Kim et al., 2001). Specific silencing of *NbRanBP1-1* caused growth defect as well as decreased disease resistance, but elongation of primary roots was not observed, thus another NbRanBP1 might be involved in the auxin response. Cho et al. (2008) reported that gene-silencing of *NbRanBP1* (*NbRanBP1-2b*) caused constitutive activation of pathogenesis related (*PR*)-genes, which is a distinct phenotype from *NbRanBP1-1-*silenced plants in this study. Phylogenetic analysis of Solanaceae RanBP1 proteins revealed that they are divided into three groups, further suggesting that they could be functionally distinctive, although Arabidopsis RanBP1s formed a separate clade in this analysis (Supplementary Figure S2).

Besides nucleo-cytoplasmic transport, Ran and RanBP1 are involved in nuclear membrane formation and spindle assembly during mitosis (Pu and Dasso, 1997; Hetzer et al., 2000; Trieselmann and Wilde, 2002; Zhang et al., 2014). Given that mammalian and yeast RanBP1 is multifunctional, growth defects in *NbRanBP1-1*-silenced plants would be caused by defects in multiple functions. There are limited numbers of reports investigating the role of plant Ran and RanBP1. Overexpression of wheat RAN1 in Arabidopsis increased primordial tissue, reduced the number of lateral roots, and stimulated hypersensitivity to exogenous auxin, same as transgenic Arabidopsis expressing antisense *RanBP1c* (Kim et al., 2001; Wang et al., 2006). Interestingly, components of Nup107-160, Nup96/Sar3, and Nup160/Sar1, have been isolated as suppressors of the Arabidopsis *auxin-resistant1* (*axr1*) mutant (Parry et al., 2006; Parry, 2014). Rice and Arabidopsis overexpressing *OsRAN2* became hypersensitive to salt, osmotic stresses and abscisic acid (Zang et al., 2010). Arabidopsis *ran1*/*ran3* double mutants, but not *ran1* or *ran3* single mutants, showed reduced tolerance to freezing stress, indicating their redundant function, while overexpression of Ran1 or Ran3 enhanced the freezing tolerance. Although mechanistic functions of plant Ran proteins were still largely unknown, previous reports showed that Ran is involved in the response to a wide range of external stresses as well as development.

In summary, we identified *N. benthamiana* RanBP1-1 as an essential factor for disease resistance. Our forward genetic screening identified NbNup75 and NbRanBP1-1 as essential genes for the resistance of *N. benthamiana* against *P. infestans* (Ohtsu et al., 2014 and this study), and indicated that NPC-mediated trafficking is a key event for induction of disease resistance. Transcriptional up-regulation of defense-related *NbWIPK* was not affected by gene silencing of *NbRanBP1-1* in the early stage of defense induction, but translation of WIPK was significantly compromised, therefore NbRanBP1 is involved in the post-transcriptional stage of defense induction (Figure 8). NbRanBP1, Nup75, and Nup160 are involved in the change of intracellular distribution of NbRan1a during defense induction, which we expect to be a key process for the induction of plant defense against biotic and possibly abiotic stresses. Much remains to be investigated regarding the specific mechanism for export of mRNAs of defense-related genes. In yeast, heat stress blocked nuclear export of most mRNAs, while mRNAs for heat-induced genes *SSA1* and *SSA4* were effectively exported to the cytoplasm (Saavedra et al., 1996). In the mammalian system, RNA binding protein HuR binds to an adenylate uridylate-rich element (ARE) in the 3’ untranslated region of certain early response genes (ERGs) to enhance the export of specific mRNAs (Gallouzi and Steitz, 2001). Such mechanisms for selective export of mRNA might be employed in plant stress responses as well.

It should by now be apparent that, more than a simple gateway, the nucleo-cytoplasmic transport represents a potent regulatory instance whereby plants regulate the response to biotic and abiotic stresses. How a seemingly general process such as nuclear transport can exert specific control on a multitude of pathways, requires further investigation of the various factors involved therein. Apart from providing important insights into our understanding of plant stress regulation, further understanding of the nucleo-cytoplasmic transport may also benefit our understanding of other plant pathways as well, as there is no reason to assume that this process is limited to plant stress alone.

## Data Availability

The datasets generated for this study can be found in GenBank, as listed in Supplementary Tables.

## Author Contributions

YM and DT designed the research. YM, MOh, YS, HM, and DT conducted the experiments. AT, MOj, and DT supervised the experiments. YM and DT wrote the manuscript. MC and DT edited the manuscript. YM, MC, IS, SC, KK, and DT contributed to the discussion and interpretation of the results.

## Conflict of Interest Statement

The authors declare that the research was conducted in the absence of any commercial or financial relationships that could be construed as a potential conflict of interest.
