# Supplementary material for: Nicotiana benthamiana RanBP1-1 Is Involved in the Induction of Disease Resistance via Regulation of Nuclear-Cytoplasmic Transport of Small GTPase Ran
Source: Front Plant Sci. 2019 Mar 8;10:222. doi: 10.3389/fpls.2019.00222 (PMC6418045; doi:10.3389/fpls.2019.00222)
Supplement: Supplementary file 1 [file Data_Sheet_1.docx]

eSupplementary Material

*Nicotiana benthamiana* RanBP1-1 is involved in the induction of disease resistance via regulation of nuclear-cytoplasmic transport of small GTPase Ran

Yuri Mizuno, Mina Ohtsu, Yusuke Shibata, Aiko Tanaka, Maurizio Camagna, Makoto Ojika, Hitoshi Mori, Ikuo Sato, Sotaro Chiba, Kazuhito Kawakita and Daigo Takemoto*

*** Correspondence:** Daigo Takemoto : dtakemo@agr.nagoya-u.ac.jp

## Supplementary Figures

**Supplementary Figure 1.** Alignment of nucleic acid sequences of *NbRanBP1-1a* (Niben101Scf00439g07002.1) and *NbRanBP1-1b* (Niben101Scf24384g00009.1). Sequences of cDNA isolated from line A4-87 is shown in green box. Sequences of cDNA used for construction of silencing vectors (NbRanBP1-1-v1, NbRanBP1-1-v2) are indicated red and blue lines, respectively.

**Supplementary Figure S2.** Phylogenetic analysis of RanBP1 from Human, *Arabidopsis thaliana* and Solanaceae plants. The tree was prepared by the neighbor-joining method (Saitou and Nei, 1987) using MacVector version 15. The scale bar corresponds to estimated amino acid substitutions per site. Numbers at the nodes indicate the percentage of 1000 bootstrap replicates that supported each labeled interior branch. Hs, *Homo sapiens*; Nb, *Nicotiana benthamiana*; At, *Arabidopsis thaliana*. Gene ID with Solyc, *Solanum lycopersicum* (Tomato); Sme, *S. melongena* (Eggplant); LOC, *Capsicum annuum* (Hot pepper), and PGSC, *S. tuberosum* (Potato).

**Supplementary Figure S3.** *Nicotiana benthamiana* plants were inoculated with TRV or TRV:RanBP1-1. Expression of *NbRanBP1-2* and *NbRanBP1-3* was assessed by qRT-PCR with gene-specific primers. Expression levels of genes were quantified relative to that of constitutively expressing *NbEF-1α*. Means ± SE (n=3). Data marked with asterisks are significantly different from control as assessed by the two-tailed student’s t test: *P < 0.05.

**Supplementary Figure S4.** Expression profiles of *Nicotiana benthamiana Ran* genes.

**(A)** Alignment of predicted amino acid sequences of NbRan1, NbRan2, NbRan3 and yeast GSP1. Conserved domains among Ran GTPases are boxed.

**(B)** Expression profiles of *NbRan1, NbRan2 and NbRan3*. Leaves of TRV-infected control *N. benthamiana* were treated with 150 nM INF1 and harvested 0 h and 12 h after treatment. Expression of genes were assessed by qRT-PCR with gene-specific primers. Expression levels of genes are quantified relative to that of constitutively expressing *NbEF-1α.* Means ±SE (n=3).

**Supplementary Figure S5.** Gene silencing of *NbRan1* and *NbRan2*.

**(A)** Location of cDNA fragments used for VIGS of *NbRan1*, *NbRan2*, and both *NbRan* genes.

**(B)** Expression of target genes in control (TRV), *NbRan1*-, *NbRan2*- or *NbRan1/2*-silenced plants were assessed by qRT-PCR with gene-specific primers. Expression levels of genes are quantified relative to that of constitutively expressing *NbEF-1α*, and gene-silencing values were shown as the fold change relative to *NbRan1* or *NbRan2* expression in control plant (TRV). Means ± SE (n=3). Data marked with asterisks are significantly different from control as assessed by the two-tailed student’s t test: *P < 0.05.

**(C)** Growth phenotype of *N. benthamiana* infected with TRV, TRV:NbRan1, TRV:Ran2 or TRV:Ran1/2. Photographs of gene-silenced plants were taken 17 days after the inoculation with *A. tumefaciens* for VIGS induction.

**(D)** (left) Distribution of poly(A) mRNA in mesophyll cells of control (TRV) and NbRan1/2-silenced *N. benthamiana* leaves probed with oligo (dT) _45_ labeled with Alexa 488. (right) Kymographs of Alexa488 fluorescence in mesophyll cells of control and *NbRan1/2*-silenced *N. benthamiana* created from images shown in left panels.

**Supplementary Figure S6.** NbRan1 and NbRan2 are partially involved in INF1-induced production of capsidiol and nuclear export of mRNA.

**(A)** Capsidiol was extracted from control, *NbRan1*- or *NbRan2*-silenced *N. benthamiana* leaves 24 and 48 h after 150 nM INF1 treatment and quantified by high performance liquid chromatography. Data are means ±SE (n=7-19). Data marked with asterisks are significantly different from control as assessed by the two-tailed student’s t test: *P < 0.05.

**(B)** Distribution of poly(A) mRNA in mesophyll cells of control (TRV), *NbRan1-* or *NbRan2-*silenced *N. benthamiana* leaves probed with oligo(dT)_45_ labeled with Alexa 488. Bar = 50 µm.

**Supplementary Figure S7.** Nuclear accumulation of NbRan1a is reduced in *Nicotiana benthamiana* epidermal cells attacked by *Phytophthora infestans.*

**(A)** Leaves of *N. benthamiana* were inoculated with *Agrobacterium tumefaciens* for expression of GFP-NbRan1a and H2B-RFP (Histone 2B for nuclei marker). Localization of GFP-NbRan1a and H2B-RFP were monitored by confocal laser scanning microscopy. Bars = 10 µm.

**(B)** Intensities of GFP and RFP fluorescence were plotted for cross section (a to b) shown in left panels. Note that GFP-NbRan1a can be detected nuclear rim as well as inside of nucleus.

**(C)** Leaves of *N. benthamiana* expressing GFP-NbRan1a and H2B-RFP were inoculated with *P. infestans,* and localization of GFP-NbRan1a and H2B-RFP were monitored by confocal laser scanning microscopy 12 h after inoculation. *P. infestans* was visualized by calcofluor white (CW) staining. a, appressorium-like swelling; z, zoosporangia. Bar = 20 µm.

**Supplementary Figure S8.** Phenotypes of *NbNup160-*silenced *Nicotiana benthamiana*.

**(A)** Growth phenotype and of *N. benthamiana* infected with TRV or TRV:Nup160. Photographs of gene-silenced plants were taken 3 weeks after the inoculation with *Agrobacterium tumefaciens* for VIGS induction.

**(B)** Expression of *NbNup160* in control (TRV) and *NbNup160*-silenced *N. benthamiana* was quantified relative to that of constitutively expressing *NbEF-1α*. Means ± SE (n=3). Data marked with asterisks are significantly different from control as assessed by the two-tailed student’s *t* test: *P < 0.05.

**(C)** Appearance of disease was categorized into 5 classes according to the severity of disease symptom described in Figure 2. Plot showing percentage of *N. benthamiana* leaves with disease symptom severities represented in the five classes, for leaves of control and *NbNup160*-silenced plants inoculated with *P. infestans* at 5 days post inoculation. At least 12 leaves from each gene-silenced plants were scored.

**(D)** Leaves of control (TRV), *NbEAS-* and *NbNup160*-silenced *N. benthamiana* were inoculated with *P. infestans*. Photographs were taken 5 days post inoculation (dpi).

**Supplementary Figure S9.** *NbNup160* is involved in nuclear export of mRNA export and elicitor-induced capsidiol production.

**(A)** Distribution of poly(A) RNA in mesophyll cells of control (TRV) and *NbNup160*-silenced *Nicotiana benthamiana* leaves detected using oligo(dT) _45_ labeled with Alexa 488. (bottom) Kymographs of Alexa488 fluorescence in mesophyll cells of control and *NbNup75*-silenced *N. benthamiana* created from images shown in top panels. Bars = 50 µm.

**(B)** Capsidiol was extracted from control or *NbNup160*-silenced *N. benthamiana* 24 h after 150 nM INF1 treatment and quantified by high performance liquid chromatography. Data are means ±SE (n=16-18). Note that no production of capsidiol was detected for water-treated samples. Data marked with asterisks are significantly different from control as assessed by the two-tailed student’s t test: *P < 0.05.

**Supplementary Figure S10.** NbNup75 and NbNup160 are involved in INF1-induced nuclear export of NbRan1a.

**(A)** Leaves of TRV-infected, *NbNup75-* or *NbNup160*-silenced *Nicotiana benthamiana* were inoculated with *Agrobacterium tumefaciens* containing expression vector for GFP-NbRan1a. Leaves expressing GFP-Ran1a were treated with water (H_2_O) or 150 nM INF1, and GFP fluorescence was observed 12 h after treatment. Bars = 50 µm.

**(B)** Appearance of fluorescence was categorized into 3 classes according to the fluorescence intensity shown in Figure 7. At least 100 cells from each silenced plants were scored for each treatment. Data marked with asterisks are significantly different from control as assessed by one-tailed Mann-Whitney U tests: **P < 0.01.

## Supplementary Tables
